# Early initiation of breastfeeding: Better practiced in primary healthcare facilities? Analysis of the 2019 National Demographic and Family Health Survey in Peru

**DOI:** 10.1371/journal.pgph.0004486

**Published:** 2025-05-09

**Authors:** Naysha Becerra-Chauca, Laura C. Altobelli

**Affiliations:** 1 Facultad de Salud Pública y Administración, Universidad Peruana Cayetano Heredia, Lima, Peru; 2 Future Generations University/Future Generations Global Network, Franklin, West Virginia, United States of America; Qatar University College of Medicine, QATAR

## Abstract

Early initiation of breastfeeding (EIBF) within the first hour of life is advocated by the World Health Organization for its numerous benefits, including emotional bonding and immunological protection for the newborn. Despite global efforts, EIBF prevalence varies significantly, with reports in Peru falling below the recommended 80%. This study investigates the association between healthcare facility level and EIBF in Peru, addressing a gap in research on the influence of health facility characteristics on EIBF rates. A cross-sectional analytical study was conducted using data from the 2019 Peru Demographic and Health Survey. We included women who had uncomplicated singleton vaginal deliveries in healthcare facilities within the previous 24 months. Poisson regression adjusted for complex sampling design was used to assess the association of interest, adjusting for socio-demographic and maternal-neonatal health variables. The analysis was made using only complete cases (no missing data). Therefore, 3,389 births met the inclusion criteria, but 3,104 were included in the analysis. It revealed that births in primary healthcare (PHC) facilities had a 10% higher prevalence of EIBF compared to secondary and tertiary facilities. Covariates associated with increased EIBF prevalence included rural residency, living in the highlands or jungle, having a newborn of normal or larger size, having a midwife as highest-ranking health professional present during delivery. Conversely, higher maternal education and wealth, as well as delivery in public hospitals financed by payroll deductions (EsSalud), were associated with lower EIBF rates. This suggests that facility-level characteristics may impact EIBF practices. Recommendations include strengthening health service policies and protocols, training for health personnel across all healthcare levels to support EIBF, and exploring stakeholder perspectives through future research.

## Introduction

Early initiation of breastfeeding (EIBF) is a strategy recommended by the World Health Organization (WHO) that involves the initiation of breastfeeding within the first hour of life of the newborn [1]. This practice offers emotional bonding benefits for the mother-infant relationship, provides immunological protection for the newborn [1], and reduces the risk of neonatal mortality [2–4] It is also significantly associated with the continuation of exclusive breastfeeding for up to six months [5]. Additionally, it is part of the quality criteria for maternal-neonatal care which includes evidence-based practices, timely and responsive care, functional health systems, effective communication, and respectful, dignified treatment to ensure both clinical effectiveness and a positive care experience [6].

Globally, the prevalence of EIBF at the country level ranges from 23.6% to 95.6% [7]. In Peru, the 2019 Demographic and Health Survey (DHS) reports EIBF prevalence below 50% [8]. However, the WHO recommends that countries achieve a minimum of 80% among term newborn mothers [9]. Multiple studies have shown an association between EIBF and socio-demographic, maternal-neonatal health, and institutional factors, such as education, wealth, residence, delivery type, parity, birth weight, skin-to-skin contact, delivery location, and staff involvement [10–14].

Few studies have evaluated the association of health facility characteristics with EIBF, providing heterogenous findings. Some studies indicate a higher likelihood of EIBF in public than private health facilities, while others mention no difference between them [10–14]. We found only one study, conducted by Karim et al. in 2018 [15] in Bangladesh, that assessed the association between level of healthcare facilities and EIBF. They found that prevalence of EIBF was higher in secondary level hospitals than in primary level facilities.

Each level of healthcare facility has different characteristics, as each is designed for addressing different health needs and interventions [16–18]. In Peru, the healthcare level is defined by the degree of specialization and technological resources available. Thus, secondary and tertiary healthcare facilities have a bigger infrastructure, offer various medical specialties and target patients with more complex conditions or pathologies, while primary healthcare (PHC) facilities are smaller, do not have as many specialties and target a broader population focusing on providing basic health services for early treatment of common illnesses with a preventive and promotional approach to family and community health [19,20].

Regarding pregnancy and childbirth care, in Peru, low risk pregnancies are managed in primary as well as secondary and tertiary healthcare facilities. However, the latter (secondary and tertiary healthcare facilities) also provide care for high-risk pregnancies with pathologies or complications. The Ministry of Health of Peru promotes EIBF through the National Norm for Comprehensive Newborn Care [21] and the National Guideline for Breastfeeding Counseling [22]. Therefore, breastfeeding should be initiated promptly regardless of the level of healthcare facility.

In PHC facilities, births are primarily attended by midwives or general practitioners, and in their absence, by nurses. In secondary and tertiary healthcare facilities, low risk births are generally attended by midwives, with obstetrician-gynecologists available if needed. Regarding care of newborn, in PHC facilities, neonatal care is primarily the responsibility of nurses and the infant is normally kept with the mother in a bassinet or in bed with her. In secondary and tertiary facilities, the presence of specialized personnel and dedicated neonatal care spaces for both normal and high-risk newborns may lead to mother-infant separation during the first few hours, depending on multiple factors. Rooming-in beyond the first few hours after birth is common in both settings.

Regarding infrastructure, PHC facilities are smaller and have less in-patient bed capacity because they tend to have less demand for childbirth services. Secondary and tertiary facilities have more or larger rooms to accommodate more women at once.

These and other characteristics such as personnel, service offerings, budgets, and other factors vary from one level to another. These differences can influence patient privacy, workload, staffing, staff training, and the quality of care, which consequently, could have an impact on EIBF. Studies have identified that a lack of privacy for women and their babies, personnel shortages, and insufficient specialized staff training could be associated with reduced EIBF [16,23–28].

PHC centers, typically characterized by lower patient volume, lighter workload for healthcare providers, and community-based care approaches, may provide a more conducive environment for supporting EIBF compared to secondary or tertiary healthcare facilities, where high patient load and resource constraints may limit personalized care. Based on these differences, this study hypothesizes that the prevalence of EIBF will be significantly higher in PHC than in secondary or tertiary healthcare facilities.

Previous research in Peru [14], has examined factors associated with EIBF using DHS data. However, the role of healthcare facility level has not been thoroughly explored. This study aims to assess specifically the association between the healthcare facility level and EIBF. Our intention is to determine whether maternal and neonatal care varies according to the level of public healthcare facility where delivery occurs.

## Methods

### Ethics statement

The researchers of the present study did not collect the data. We utilized the DHS database, which is publicly accessible, and the data were collected anonymously. The present study was approved by the Institutional Ethics Committee of “Universidad Peruana Cayetano Heredia” Certificate Nº “608 - 36 – 20”.

### Data

We conducted a cross-sectional analytical study based on secondary analysis of the 2019 Peru Demographic and Health Survey (DHS) carried out by the Peruvian National Institute of Statistics and Informatics (INEI) [8]. This national-level survey was conducted by the National Institute of Statistics and Informatics. The sampling design was a two-stage, probabilistic, stratified, and independent process, stratified by department and further subdivided into urban and rural strata. In the first stage, primary sampling units (PSUs), typically corresponding to census enumeration areas (EAs), were selected with probability proportional to size, based on the population from the most recent national census. In the second stage, a fixed number of households within each selected PSU were systematically sampled. Sampling weights were applied to account for unequal probabilities of selection [29].

Cases included in the current study were: women with uncomplicated singleton pregnancies, with vaginal delivery, within the previous 24 months at a public sector healthcare facility of either the Peruvian Ministry of Health (MOH) or the Peruvian Social Security Institute (EsSalud in Spanish). The inclusion of women with infants that were live-born within the prior 24 months is in accordance with the WHO denominator for EIBF [30]. Private sector facilities were excluded because, in the DHS dataset, they are categorized under broad labels, without a clear distinction between levels of care. To ensure a meaningful comparison across facility levels, we restricted our analysis to public establishments, where such differentiation is explicitly available. Excluded cases were those experiencing any childbirth complications as reported in the DHS survey (prolonged labor, excessive bleeding, fever with vaginal bleeding, seizures, others), as literature suggests that in these situations, the initiation of breastfeeding would be postponed to safeguard the mother’s life [6,17,31]. Additionally, maternal complications could confound the association between healthcare facility level and EIBF. Since secondary and tertiary facilities manage high-risk pregnancies, while primary-level facilities handle low-risk deliveries and refer complicated cases, including maternal complications could introduce systematic differences between facility levels. While newborn complications were considered in our conceptual model, this information was not available in the dataset and could not be included as an exclusion criterion.

We used the entire available sample that met our inclusion criteria; therefore, a formal power calculation was not necessary.

Data collection was conducted by a team of household interviewers selected and trained by INEI, following a structured questionnaire to directly interview selected women each selected household, using electronic tablets to register responses.

### Variables

As the outcome variable, EIBF was defined as the newborn being placed on the mother’s breast within the first hour of birth [1]. This information was derived from the question: “How long after the birth of (NAME) did you begin to give the breast?” If the mother’s response was less than one hour, it was recorded as Immediately = 0. If her response was less than 24 hours, it was recorded as Hours = 101–123. If her response was more than 24 hours, it was recorded as Days = 201–2XX. For the purposes of this study, the variable was categorized into a dichotomous variable. A data entry of 0 was recoded as “YES” (with EIBF), while data entries of more than 100 were recoded as “NO” (without EIBF).

The exposure variable was the level of public sector healthcare facility where the birth was attended, obtained from the question “Where did you give birth to (NAME)?” The possible responses were: Home; MoH Hospital; EsSalud Hospital; Armed Forces and Police Hospital; MoH Health Center; MoH Health Post; EsSalud Polyclinic/Center/Post; Municipal Hospital/Other; Private Clinic; Private Medical Office; Midwive’s House; Non-Governmental Organization (NGO) Clinic/Post; Church Hospital/Other; Other (specify).

For the purposes of this study, we focused exclusively on births that occurred in public sector facilities managed by the Ministry of Health (MoH) or EsSalud. Responses indicating “Health Center”, “Post”, or “Polyclinic” (MoH Health Center, MoH Health Post and EsSalud Polyclinic/Center/Post) were grouped into the category “Primary healthcare center”. In contrast, responses indicating “Hospitals” (MoH Hospital and EsSalud Hospital) were categorized as “Secondary and tertiary healthcare centers”. This recategorization reflects the organization of Peru’s health system, where health posts and health centers serve as primary care facilities, while hospitals provide secondary and tertiary care.

The covariates included were: mother’s age group (15–24, 25–35, 36–49), place of residence (urban, rural), mother’s educational level (none/any primary, any secondary, any higher), wealth index (very poor, poor, medium, rich, very rich), region of residence (coastal, highlands, jungle), mother’s ethnicity (white/mestizo/other, Quechua/Aymara/Amazonian/other, indigenous, Afrodescendant), mother’s marital status (lives with partner, does not live with partner), received breastfeeding counseling (yes, no), number of prenatal check-ups (8 or more, less than 8), birth weight ≤2500 gm. (yes, no), birth size (very small/small, normal/large/very large), birth order of child (1st child, 2nd child, 3rd or higher child), highest-ranking health professional present during delivery (medical doctor, midwife), and healthcare facility financing (MoH, EsSalud).

### Statistical analysis

We conducted the analysis using statistical software STATA 16 for Windows (StataCorp, College Station, TX, USA). To adjust all the analyses for the complex sampling design, we applied the data weighting factors provided in the data set (V005 = women factor) and considering the conglomerate (V001) and stratum (V022) of the sample. This information was reported in the Technical Data Sheet of the 2019 Peru DHS [32].

All variables, including the exposure and covariates, were described according to the level of the public sector healthcare facility where the birth occurred (primary level vs. secondary/tertiary level). Chi-squared tests were performed to assess differences in these variables between facility-level groups.

An initial descriptive univariate analysis was applied to categorical variables. Bivariate relationships were assessed between EIBF and healthcare facility level where childbirth occurred, and covariates. A Poisson regression was used for calculating prevalence ratio (PR) in unadjusted and adjusted models. Given that the outcome variable has a prevalence close to 50%, logistic regression was not used, as it may overestimate associations and yield odds ratios that are difficult to interpret. Poisson regression provides unbiased and easily interpretable prevalence ratio estimates [33,34], making it a suitable alternative for common outcomes in epidemiological studies. Standard errors were estimated using Taylor series linearization (first-order approximation) to account for the complex survey design, including clustering at the primary sampling unit level. This method was implemented via the svy package in Stata v.16, which provides robust variance estimation for weighted survey data [35]. Sampling weights were applied in all analyses to correct for unequal selection probabilities and non-response, following the guidelines specified in the DHS technical documentation [32].

For the adjusted model, we developed a framework using a Directed Acyclic Graph (DAG) (S1 Text and S1 Fig) based on variables previously associated with EIBF reported in previous studies [14,36–40]. Anticipated variables included mother’s age, mother’s ethnicity, wealth index, birth size, region of residence, place of residence, low birth weight, mother’s educational level, healthcare facility financing, number of prenatal check-ups, and birth order of child.

It is important to note that only complete cases were included in the multivariable analysis; observations with missing data were excluded to ensure robust and unbiased estimates in the adjusted models.

To assess the potential modification effect of the variables “highest-ranking health professional present during delivery,” “type of healthcare facility financing,” and “place of residence,” interaction analyses were conducted using an adjusted Wald test. PR was calculated for each subgroup when an interaction was found.

## Results

A total of 21,154 women aged 15 to 49 with a live birth in the previous five years responded to the DHS survey questionnaire on pregnancy, childbirth, postpartum, and breastfeeding. Only 4,521 of them met the inclusion criteria. An additional 1,132 cases were excluded due to complications during childbirth. Ultimately, 3,389 births were included in the analysis.

Among the study cases, 74.5% initiated breastfeeding within the first hour of childbirth. As to place of birth, 30.7% were attended in PHC facilities, while 69.3% were attended in secondary or tertiary healthcare facilities. The majority of study mothers were aged 25 to 34, lived in urban areas, on the coast, and had completed secondary education. About 53.2% of mothers identified as white/mestizo/other, and 83.9% were partnered**.** Regarding EIBF and healthcare level, 83.5% of study mothers who attended PHC facilities-initiated breastfeeding within the first hour of birth, compared to 70.6% of those who delivered in secondary or tertiary facilities. Differences according to the level of healthcare facility where birth occurred are shown in Table 1.

**Table 1:** Characteristics of included sample (*).

		All n = 3389 (100%)	Primary healthcare facilities n = 1083 (30.7%)	Secondary or tertiary facilities n = 2306 (69.3%)	p value
**Early initiation of breastfeeding (EIBF)**	**Yes**	2,601 (74.5%)	935 (83.5%)	1,666 (70.6%)	<0.01
	**No**	788 (25.5%)	148 (16.5%)	640 (29.4%)	
**Mother’s age**	**15 - 24**	1,153 (33.7%)	400 (36.3%)	753 (32.6%)	0.08
	**25 - 34**	1,517 (45.5%)	477 (45.6%)	1,040 (45.5%)	
	**35 - 49**	709 (20.7%)	204 (18.2%)	505 (21.9%)	
	**Missing**	10	2	8	
**Place of residence**	**Rural**	1,106 (30.4)	529 (46.5%)	577 (23.3%)	<0.01
	**Urban**	2,283 (69.6)	554 (53.5%)	1,729 (76.7%)	
**Region of residence**	**Costal**	1,236 (48.9)	222 (31%)	1,014 (56.8%)	<0.01
	**Highlands**	1,192 (30.2)	506 (40.1%)	686 (25.5%)	
	**Jungle**	961 (20.9)	355 (28.2%)	606 (17.7%)	
**Mother’s educational level**	**None/any Primary**	695 (20.8)	336 (30.1%)	359 (16.6%)	<0.01
	**Any secondary**	1,773 (52.1)	581 (52.9%)	1,192 (51.8%)	
	**Any higher**	921 (27.1)	166 (17.03%)	755 (31.6%)	
**Wealth index**	**Very poor**	1,093 (28.8)	544 (46.5%)	549 (20.1%)	<0.01
	**Poor**	1,034 (28.4)	334 (31.1%)	700 (27.2%)	
	**Medium**	647 (20.2)	146 (13.2%)	501 (23.3%)	
	**Rich**	416 (14.6)	42 (6.1%)	374 (18.4%)	
	**Very Rich**	199 (7.9)	17 (3%)	182 (10.1%)	
**Mother’s ethnicity**	**White/mestizo/other**	1,557 (53.2)	395 (45.1%)	1,162 (56.9%)	<0.01
	**Quechua/Aymara/ Amazonian/other**	1,249 (32.9)	520 (41.1%)	729 (29.2%)	
	**Afro descendant**	367 (13.9)	114 (13.8%)	253 (14%)	
	**Missing**	216	54	162	
**Mother’s marital status**	**Lives with partner**	2,846 (84%)	930 (85%)	1,916 (83.4%)	0.35
	**Lives without partner**	543 (16%)	153 (15%)	390 (16.6%)	
**Received breastfeeding counseling**	**Yes**	2,578 (75.2)	864 (78.6%)	1,714 (73.7%)	0.019
	**No**	811 (24.8)	219 (21.5%)	592 (26.3%)	
**Number of prenatal check-ups**	**8 or more**	2,442 (71.7)	755 (62.9%)	1687 (72.9%)	0.07
	**Less than 8**	940 (28.3)	323 (31.1%)	617 (27.1%)	
	**Missing**	7	5	2	
**Low birth weight**	**Yes**	139 (4.3)	42 (4.2%)	97 (4.3%)	0.91
	**No**	3,231 (95.7)	1,033 (95.8%)	2,198 (95.7%)	
	**Missing**	19	8	11	
**Birth size**	**Very small/small**	699 (20.4)	232 (20.8%)	467 (20.3%)	0.8
	**Normal/large/very large**	2,689 (79.6)	851 (79.2%)	1,838 (79.7%)	
	**Missing**	1	0	1	
**Birth order of child**	**1st child**	1,050 (31.6)	270 (25.9%)	780 (34.1%)	<0.01
	**2nd child**	1,040 (31.1)	345 (32.3%)	695 (30.5%)	
	**3rd or higher child**	1,299 (37.4)	468 (41.8%)	831 (35.4%)	
**Highest-ranking health professional present during delivery**	**Medical doctor**	1,540 (52.8)	389 (41.2%)	1,151 (57.9%)	<0.01
	**Midwife**	1,811 (47.2)	667 (58.9%)	1,144 (42.1%)	
	**Missing**	38	27	11	
**Healthcare facility financing**	**MoH - MINSA**	2,817 (82.1)	1,073 (98.8%)	1,744 (74.8%)	<0.01
	**EsSalud**	572 (17.9)	10 (1.2%)	562 (25.2%)	

(*) Weighted analysis based on weighting factor reported by DHS survey.

**Fig 1:**
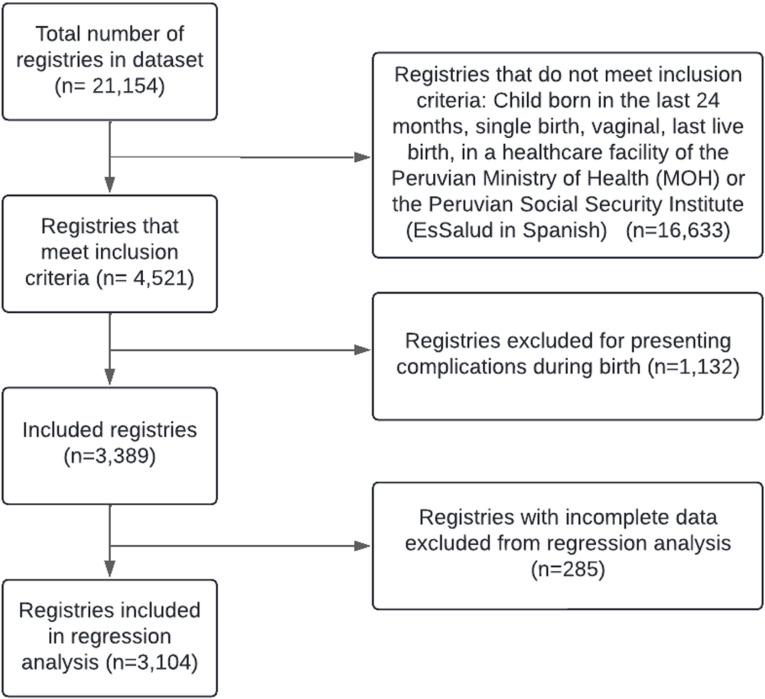
Flow diagram of participant selection.

Of the 3,389 observations in the dataset, 285 cases (8.41%) were excluded from the Poisson regression analyses due to missing data. The distribution of missingness was as follows: 6.4% of observations were missing data on mothers’ ethnicity, and 1.12% on highest-ranking health professional present during delivery. The remaining covariates with missing data (Low birth weight, Birth size, Age, and Number of prenatal check-ups) had less than 1% missing data. Given the low proportion of excluded cases, it is unlikely that this missingness introduced significant bias. A complete case analysis was performed, resulting in a final analytical sample of 3,104 cases (Fig 1).

In the unadjusted analysis, EIBF prevalence was higher in PHC facilities (PR 1.17 95% CI [1.11 to 1.24]). Other associated covariates with increased prevalence of EBFI were, living in rural areas (PR 1.13, 95% CI [1.08 to 1.20]), living in the highlands (PR 1.15, 95% CI [1.08 to 1.22]) or jungle (PR 1.26, 95% CI [1.19 to 1.34]), having a newborn with a normal, large, or very large size (PR 1.11, 95% CI [1.03 to 1.20]) and having a midwife as highest-ranking health professional present during delivery (PR 1.07, 95% CI [1.02 to 1.14]) increase the prevalence of EIBF. Conversely, having a wealth index higher than poor, a mother with higher education level, low-weight birth and if the healthcare facility is financed by EsSalud, were associated with a lower prevalence of EIBF. Mothers’ age, having a secondary education level, identifying as Quechua or Aimara, indigenous or Afro-descendant, number of prenatal appointments, order of birth, marital status, receiving breastfeeding counseling were not found to be associated to EIBF (Table 2).

**Table 2:** Association of variables with early initiation of raw and adjusted breastfeeding.

Variable		cPR^a^ [95% CI] (*) N = 3104^c^	aPR^b^ [95% CI] (*) N = 3104^c^
Healthcare facility level	Primary healthcare facilities	1.17 [1.11 - 1.24]	1.10 [1.04 - 1.16]
	Secondary or tertiary facilities	Ref	Ref
Mother’s age	15 - 24	Ref	Ref
	25 - 35	0.95 [0.90 - 1.01]	0.95 [0.88 - 1.02]
	36 - 49	0.93 [0.86 - 1.01]	0.92 [0.83 - 1.03]
Place of residence	Urban	Ref	Ref
	Rural	1.13 [1.08 - 1.20]	1.05 [0.99 - 1.12]
Mother’s educational level	None/any Primary	Ref	Ref
	Any secondary	0.98 [0.91 - 1.04]	1.01 [0.94 - 1.09]
	Any higher	0.86 [0.79 - 0.93]	0.95 [0.86 - 1.06]
Wealth index	Very poor	Ref	Ref
	Poor	0.94 [0.88 – 1.00]	1.02 [0.96 – 1.10]
	Medium	0.90 [0.84 - 0.97]	1.06 [0.96 – 1.17]
	Rich	0.83 [0.75 - 0.92]	1.03 [0.91 – 1.16]
	Very Rich	0.83 [0.73 - 0.95]	1.09 [0.93 – 1.27]
Region of residence	Costal	Ref	Ref
	Highlands	1.15 [1.08 - 1.22]	1.10 [1.02 - 1.19]
	Jungle	1.26 [1.19 - 1.34]	1.20 [1.12 - 1.29]
Mother’s ethnicity	White/mestizo/other	Ref	Ref
	Quechua/Aymara/Amazonian/other	1.06 [0.99 - 1.12]	1.02 [0.95 - 1.08]
	Afrodescendant	1.06 [0.98 - 1.15]	1.05 [0.97 - 1.14]
Number of prenatal check-ups	8 or more	Ref	Ref
	Less than 8	1.03 [0.97 - 1.10]	1.04 [0.98 - 1.10]
Low birth weight	No	Ref	Ref
	Yes	0.78 [0.64 – 0.95]	0.82 [0.67 – 1.00]
Birth size	Very small/small	Ref	Ref
	Normal/large/very large	1.11 [1.03 - 1.20]	1.07 [0.99 - 1.16]
Birth order of child	1st child	Ref	Ref
	2nd child	1.05 [0.98 - 1.13]	1.06 [0.98 - 1.15]
	3rd or higher child	1.06 [0.99 - 1.13]	1.07 [0.97 - 1.18]
Healthcare facility financing	MoH - MINSA	Ref	Ref
	EsSalud	0.83 [0.75 - 0.91]	0.91 [0.83 - 1.01]
Mother’s marital status	Does not live with partner	Ref	–
	Lives with partner	1.01 [0.94 - 1.09]	–
Received breastfeeding counseling	No	Ref	–
	Yes	1.03 [0.97 - 1.10]	–
Highest-ranking health professional present during delivery	Medical doctor	Ref	–
	Midwife	1.07 [1.02 - 1.14]	–

(*) A Poisson regression was used and standard errors were estimated using Taylor series linearization (first-order approximation) to account for the complex survey design, including clustering at the primary sampling unit level. We applied the data weighting factors provided in the data set (V005 = women factor) and considering the conglomerate (V001) and stratum (V022) of the sample.

^a^cPR = Crude prevalence ratios.

^b^aPR = Adjusted prevalence ratios accounting for all variables included in the DAG.

^c^Analysis conducted on complete cases only; observations with missing data were excluded.

The adjusted model developed using DAG methodology based on literature review [14,36–40] included mother’s age, mother’s ethnicity, wealth index, region of residence, place of residence, low birth weight, birth size, mother’s educational level, healthcare facility financing, number of prenatal check-ups, and birth order of child. It was found that the effect size of healthcare facility where birth occurred on EIBF decreased but remained significant (PR of 1.10 95% CI [1.04 to 1.16]) (Table 2)

The interaction analysis did not find that the highest-ranking health professional present during delivery (p = 1.00), place of residence (p = 0.19), or healthcare facility financing (p = 0.62) modified the effect of the complexity level of the healthcare facility on EIBF. The complete Stata output for the interaction test (Wald tests) are presented in S2 Fig.

## Discussion

This study found that the adjusted prevalence of EIBF is 1.10 times (or 10%) higher in PHC facilities as compared to that in secondary or tertiary facilities.

The study by Karim et al. 2018 [15], conducted in Bangladesh, found a significant relationship as well, but in the opposite direction. In that study, giving birth in district hospitals (higher-level facilities) increased the likelihood of EIBF compared to sub-district hospitals (lower-level facilities). This could be explained by the reduced availability of staff [41], and the lack of privacy observed in lower-level facilities in that region [10]. It is worth noting that the Bangladesh study had a non-probabilistic sample of 249 observed deliveries, which is less than half of their calculated sample size. Therefore, their results may not accurately reflect the reality of that hospital but only of the included group of individuals.

In contrast to the findings of Karim et al. 2018 [15], our results suggest that women attended in primary level PHC facilities are more likely to initiate breastfeeding within the first hour of childbirth. This indicates that differences in health care level characteristics could be associated with disparities in access to EIBF.

Differences in service demand and capable human resource availability could also explain the results. A higher-level healthcare facility might have a greater demand for childbirth care compared to lower-level facilities [42], potentially leading to staff exhaustion and shortages [25]. Thus, human resource shortages due to greater demand could be a barrier to EIBF by hindering the necessary support and guidance, as reported by Kinshella et al. 2021 [28] since immediate post-natal encouragement to breastfeed by health workers and getting help in positioning babies are associated with increased EIBF [43].

The availability of specialists also varies across facility levels. In PHC facilities, deliveries are typically attended by midwives and general practitioners, whereas secondary and tertiary care facilities include medical specialists such as gynecologists and neonatologists. Wieczorek et al. 2016 [23] found that midwives tend to adopt a more empowerment-based approach to breastfeeding, whereas nurses and physicians often take a more directive role, which may impact EIBF initiation. However, our interaction analysis found no significant EIBF differences between deliveries attended by doctors or midwives, suggesting that other factors may be more influential.

Beyond staffing and provider approach, institutional practices also play a role in EIBF disparities. Sobel et al. [44] observed that unnecessary suctioning performed by resuscitation-trained specialized pediatric staff was associated with lower EIBF rates, while its absence correlated with longer breastfeeding durations. This suggests that institutional practices, may also hinder EIBF. Routine newborn interventions, such as early bathing, mother-newborn separation, and formula feeding, have been linked to delayed breastfeeding initiation [45].

Additionally in Peru, public sector PHC facilities have fewer beds and smaller spaces for childbirth services, which could lead to greater privacy during care. On the other hand, public sector secondary and tertiary facilities have more beds and larger spaces, potentially resulting in less privacy during care. While our study did not directly measure privacy, previous research has suggested that the lack of privacy may decrease the likelihood of EIBF, as noted by Shobo et al. 2020 [27], Karim et al. 2018 [15], and Kinshella et al. 2021 [28]. Although these studies were conducted in different regions, the importance of privacy in maternal care has been highlighted globally, suggesting that it could be a relevant factor in our setting as well.

We found that the prevalence of EIBF among women aged 15–49 years who had a live birth of a singleton child with vaginal delivery without maternal complications in the previous 24 months, is 74.5%. While this percentage is higher than the national average of 49.8% [8], this difference is expected given that our study population consists exclusively of uncomplicated vaginal births, which are more likely to facilitate EIBF. However, this prevalence still falls below the WHO’s recommended minimum of 80% [9]. In PHC facilities, the similarly unadjusted EIBF prevalence was 83.5%, surpassing the recommended percentage, while in secondary and tertiary facilities this fell to 70.6%.

We observed that 30.7% of our sample of study mothers attended PHC facilities for childbirth services. Significant differences were identified between the levels of healthcare facilities in terms of place of residence, region, mother’s educational level, wealth index, ethnicity, birth order of the child, and healthcare facility financing. The adjusted analysis following the DAG Model included all these variables, except for the highest-ranking health professional present during delivery, as this variable is considered a mediator. It would be important to identify the factors influencing the choice between PHC services and higher levels of care for childbirth, but such an evaluation is beyond the scope of this study.

The interaction analyses did not reveal any potential effect modification by the type of personnel attending the delivery, type of healthcare facility financing, or place of residence.

### Limitations

The study is not devoid of limitations. Since it is a cross-sectional study, we cannot establish causality, only an association between the variables of interest. There is a possibility of recall bias since the data were collected through self-reports from women [42]. Given that our inclusion criteria restricted the sample to women who had given birth in the last 24 months, this recall period is limited to two years. The analysis was constrained to the variables available in the dataset, potentially leading to residual confounding. We could not measure newborn-related interventions, such as formula feeding due to its easy availability in certain healthcare facilities and the high workload [46,47] which have been shown to be negatively associated with EIBF [44,45]. Similarly, we could not measure outdated care practices such as separating newborns from their mothers and routine provision of oral fluids to the newborn that could impose barriers to EIBF [48,49]. This residual confounding could either attenuate or exaggerate the effect size, which must be considered when interpreting the results. Additionally, the possibility of selection bias should be taken into account, which we have tried to control through multiple regression; however, there may be other variables influencing the choice of healthcare facility level for childbirth and altering the results. Furthermore, while we excluded women with those experiencing any childbirth complications as reported in the DHS survey, we intended to exclude births with neonatal complications as well, but data on this variable were not available. This is a limitation, as neonatal complications could impact EIBF and introduce bias. Finally, the use of complete case analysis for the multivariable models, resulted in the exclusion of 8.41% of the dataset due to missing values. While this loss of data reduced the overall sample size, we consider the impact on the validity of our findings to be minimal, as the proportion of excluded cases was small and unlikely to introduce significant bias. A strength of this study was the use of data collected from a nationally representative sample of women.

## Conclusion

These findings suggest that giving birth in a PHC facility is likely associated with a higher prevalence of EIBF compared to a secondary or tertiary facility. However, the reasons behind these differences remain unclear and warrant further investigation.

In light of these findings, strategies to strengthen institutional policies, protocols, and health personnel training to support EIBF should take into account the barriers that may vary by facility level. This should necessarily include efforts to reduce the influence of infant formula industries on protocols for the establishment of successful breastfeeding for newborns. In regard to PHC facilities, strategies should be implemented for improving access of the population to first-level delivery care and timely referral to higher level care if needed, improving maternal-neonatal care capacity at that level, establishing and maintaining maternity waiting homes for dispersed populations, incorporating specialized trained community health workers to monitor and refer mothers for timely first-level maternity care, etc.

Finally, further research is necessary to identify the specific factors contributing to disparities in EIBF prevalence. Prospective and qualitative studies are recommended to explore the perspectives of stakeholders involved in EIBF. Understanding these factors is essential for designing effective interventions tailored to each level of care.

## Supporting information

S1 Text Directed Acyclic Graph (DAG) illustrating the relationship between healthcare facility level and early initiation of breastfeeding (EIBF). (PDF)

S1 Fig Directed Acyclic Graph (DAG) illustrating the relationship between healthcare facility level and early initiation of breastfeeding (EIBF). (PDF)

S2 Fig Stata output for the interaction test (Wald tests). (PDF)
