# Supplementary material for: Early initiation of breastfeeding: Better practiced in primary healthcare facilities? Analysis of the 2019 National Demographic and Family Health Survey in Peru
Source: PLOS Glob Public Health. 2025 May 9;5(5):e0004486. doi: 10.1371/journal.pgph.0004486 (PMC12063802; doi:10.1371/journal.pgph.0004486)
Supplement: S1 Text — (PDF) [file pgph.0004486.s001.pdf]

Supporting information

Early initiation of breastfeeding: Better practiced in primary healthcare facilities? Analysis of the 2019 National Demographic and Family Health Survey in Peru.

Short title: "Early breastfeeding initiation by level of healthcare facility in Peru"

Naysha Becerra-Chauca[1](*), Laura C Altobelli [1,2]

[1] Facultad de Salud Pública y Administración, Universidad Peruana Cayetano Heredia, Lima, Peru

[2] Future Generations University/Future Generations Global Network, Frankin, West Virginia, United States of America

(*) E-mail address: naysha.becerra@upch.pe

NB was responsible of the conceptualization of the research question, methodology, investigation, formal analysis, writing – original draft preparation and review & editing. LA was responsible for methodology, supervision, validation and writing – review & editing.

S1 Text. Directed Acyclic Graph (DAG) illustrating the relationship between healthcare facility level and early initiation of breastfeeding (EIBF).

To establish a conceptual framework for analyzing the determinants of Early Initiation of Breastfeeding (EIBF), we constructed a Directed Acyclic Graph (DAG) using Daggity. The variables included in the DAG were selected based on a combination of empirical evidence and expert judgment, following best practices in causal inference modeling [1,2].

Sources of Variables in the DAG

The variables included in the DAG were chosen based on previous studies identifying factors associated with EIBF. Whenever available, we prioritized evidence from Peruvian populations to align with the study context. The primary references supporting the inclusion of these variables are:

- “mother’s ethnicity”[3]; “region of residency”[3,4]; “Place of residence (urban/rural)” [3,4]; “Mother’s age” [3], “mother’s education level”[3,5,6], “wealth index” [3],” number of prenatal check-ups”[3, 6]; “Birth size” [3], “low birth weight”[7], “gestational age”(*); “Healthcare facility financing”(*); “Birth order of the child”[3, 8]; “Complication in newborn”(*); “Complications during childbirth”[9]; “Type of pregnancy” [3]; “Highest-ranking health professional present during delivery”(*); Type of health professional caring for newborn”(*); “breastfeeding counseling” [3]; skin-to-skin contact”[10]; “mother’s marital status”(*).

Although some of these variables are well-documented as determinants of EIBF, there is no direct empirical evidence of the impact of others (*) on it. Additionally, to our knowledge, there is no evidence on how all of them are associated to the place where delivery occurs: primary vs. secondary/tertiary level healthcare facilities. However, based on expert judgment, a plausible causal pathway exists linking these factors to the exposure and outcome. Given this gap in the literature, we incorporated these variables into the DAG to reflect a hypothesized causal structure following best practices in causal inference modeling [1, 2].

Minimally Sufficient Adjustment Set

To determine the minimally sufficient adjustment set, we used Daggity, which identified the following variables:

- Birth order of child
- Birth size
- Complication in newborn*
- Complications during childbirth*
- Healthcare facility financing
- Low birth weight
- Mother's educational level
- Mother’s age
- Number of prenatal check-ups
- Place of residence
- Prenatal care services facilities' level*
- Type of pregnancy*
- Wealth index
- Gestational age*

Due to methodological and data availability constraints:

1. “*Complications during childbirth*”, and “*Type of pregnancy*” were used as exclusion criteria, as the study included only singleton, uncomplicated deliveries. These variables, therefore, do not vary within the included sample.
2. “*Prenatal care services facilities' level*”, “*gestational age*” and “*Complication in newborn*” were not available in the dataset, preventing their inclusion in the adjustment set.

However, in our final model, we additionally included “*mother’s ethnicity*” and “*region of residence*”, despite not being part of the minimally sufficient set suggested by Daggity.

The inclusion of these variables is justified based on **epidemiological and theoretical considerations**:

- “Mother’s ethnicity” has been linked to disparities in healthcare access, cultural norms regarding breastfeeding [3], and could potentially be linked to differences in facility-based delivery preferences.
- “Region of residence” influences healthcare facility accessibility, resource allocation, and quality of maternal care, which may indirectly affect both place of delivery and early initiation of breastfeeding [3, 4].

While Daggity identifies a minimal adjustment set, observational studies often include additional confounders to increase model robustness and reduce residual confounding [11]. Therefore, we opted to retain these variables to ensure a more comprehensive control of confounding and enhance the validity of our estimates.

The final set of variables included in the model were: mother’s age, mother's ethnicity, wealth index, region of residence, place of residence, low birth weight, birth size, mother's educational level, healthcare facility financing, number of prenatal check-ups, and birth order of child.

Rationale for Excluding Certain Variables from the Model

Following best practices in causal inference, mediators and highly correlated variables were not included in the adjustment set:

- Mediators (*highest-ranking professional in delivery*): Adjusting for mediators can introduce overadjustment bias, distorting the effect estimation of [1,12].
- Highly correlated variables (*skin-to-skin contact*): *Skin-to-skin contact* occurs almost simultaneously with EIBF, making it inappropriate for inclusion as a confounder. Adjusting for this variable could introduce time-dependent bias and potentially lead to reverse causation [12].

Conclusion

The DAG presented in the S1 Fig. reflects the conceptual framework used to analyze the determinants of EIBF while considering the limitations imposed by data availability. Although we acknowledge the lack of direct empirical evidence on how these variables influence healthcare facility complexity, we incorporated expert judgment to construct a plausible causal pathway. Our final adjustment set, determined by Daggity and the authors expertise, was constrained by the available data, but adheres to best practices in causal inference to ensure methodological robustness.

References

1. Greenland S, Pearl J, Robins JM. Causal diagrams for epidemiologic research. Epidemiology (Cambridge, Mass). 1999;10(1):37-48. Epub 1999/01/15. PubMed PMID: 9888278.
2. Williamson EJ, Aitken Z, Lawrie J, Dharmage SC, Burgess JA, Forbes AB. Introduction to causal diagrams for confounder selection. Respirology (Carlton, Vic). 2014;19(3):303-11. Epub 2014/01/23. doi: 10.1111/resp.12238. PubMed PMID: 24447391.
3. Hernández-Vásquez A, Chacón-Torrico H. Determinants of early initiation of breastfeeding in Peru: analysis of the 2018 Demographic and Family Health Survey. Epidemiology and health. 2019;41:e2019051. Epub 2020/01/22. doi: 10.4178/epih.e2019051. PubMed PMID: 31962038; PubMed Central PMCID: PMCPMC6976726.
4. Nkoka O, Ntenda PAM, Kanje V, Milanzi EB, Arora A. Determinants of timely initiation of breast milk and exclusive breastfeeding in Malawi: a population-based cross-sectional study. International breastfeeding journal. 2019;14:37. Epub 2019/08/21. doi: 10.1186/s13006-019-0232-y. PubMed PMID: 31428184; PubMed Central PMCID: PMCPMC6697947.
5. Ahmed AE, Salih OA. Determinants of the early initiation of breastfeeding in the Kingdom of Saudi Arabia. International breastfeeding journal. 2019;14:13. Epub 2019/04/16. doi: 10.1186/s13006-019-0207-z. PubMed PMID: 30984282; PubMed Central PMCID: PMCPMC6444675.
6. Islam MA, Mamun A, Hossain MM, Bharati P, Saw A, Lestrel PE, et al. Prevalence and factors associated with early initiation of breastfeeding among Bangladeshi mothers: A nationwide cross-sectional study. PloS one. 2019;14(4):e0215733. Epub 2019/04/26. doi: 10.1371/journal.pone.0215733. PubMed PMID: 31022237; PubMed Central PMCID: PMCPMC6483221.
7. Farkas C, Girard LC. Breastfeeding initiation and duration in Chile: understanding the social and health determinants. Journal of epidemiology and community health. 2019;73(7):637-44. Epub 2019/03/15. doi: 10.1136/jech-2018-211148. PubMed PMID: 30867222.
8. Chipojola R, Lee GT, Chiu HY, Chang PC, Kuo SY. Determinants of breastfeeding practices among mothers in Malawi: a population-based survey. International health. 2020;12(2):132-41. Epub 2019/07/12. doi: 10.1093/inthealth/ihz034. PubMed PMID: 31294780; PubMed Central PMCID: PMCPMC7057137.
9. Takahashi K, Ganchimeg T, Ota E, Vogel JP, Souza JP, Laopaiboon M, et al. Prevalence of early initiation of breastfeeding and determinants of delayed initiation of breastfeeding: secondary analysis of the WHO Global Survey. Sci Rep. 2017;7:44868. Epub 2017/03/23. doi: 10.1038/srep44868. PubMed PMID: 28322265; PubMed Central PMCID: PMCPMC5359598.
10. Giang HTN, Hieu LTM, Duy DTT, Phuong MT, Trung TD. The effect of skin-to-skin contact on early initiation of breastfeeding among women in Vietnam. Pediatrics and neonatology. 2024;65(4):359-64. Epub 2023/12/07. doi: 10.1016/j.pedneo.2023.07.007. PubMed PMID: 38057258.
11. VanderWeele TJ, Shpitser I. A new criterion for confounder selection. Biometrics. 2011;67(4):1406-13. Epub 2011/06/02. doi: 10.1111/j.1541-0420.2011.01619.x. PubMed PMID: 21627630; PubMed Central PMCID: PMCPMC3166439.
12. Schisterman EF, Cole SR, Platt RW. Overadjustment bias and unnecessary adjustment in epidemiologic studies. Epidemiology (Cambridge, Mass). 2009;20(4):488-95. Epub 2009/06/16. doi: 10.1097/EDE.0b013e3181a819a1. PubMed PMID: 19525685; PubMed Central PMCID: PMCPMC2744485.
