# Supplementary material for: Early initiation of breastfeeding: Better practiced in primary healthcare facilities? Analysis of the 2019 National Demographic and Family Health Survey in Peru
Source: PLOS Glob Public Health. 2025 May 9;5(5):e0004486. doi: 10.1371/journal.pgph.0004486 (PMC12063802; doi:10.1371/journal.pgph.0004486)

S1 Fig. Directed Acyclic Graph (DAG) illustrating the relationship between healthcare facility level and early initiation of breastfeeding (EIBF)

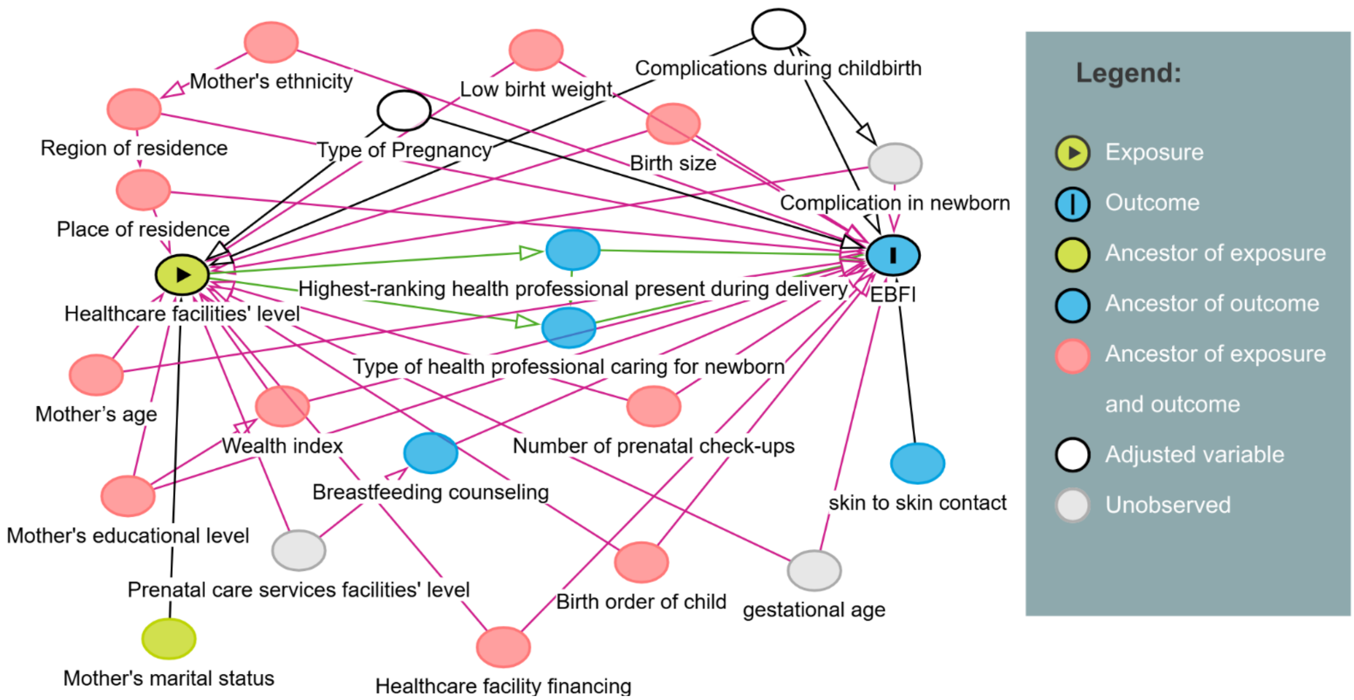

Supplement: S1 Fig — (PDF) [file pgph.0004486.s002.pdf]
