# Supplementary material for: Early initiation of breastfeeding: Better practiced in primary healthcare facilities? Analysis of the 2019 National Demographic and Family Health Survey in Peru
Source: PLOS Glob Public Health. 2025 May 9;5(5):e0004486. doi: 10.1371/journal.pgph.0004486 (PMC12063802; doi:10.1371/journal.pgph.0004486)
Supplement: S2 Fig — (PDF) [file pgph.0004486.s003.pdf]

S2 Fig. Stata output for the interaction test (Wald tests)

S2.1 Interaction test for the variable 'Highest-ranking health professional present during delivery”

```
. testparm i.Nivel_Complejidad_Parto2#i.personal_atendio_parto2

Adjusted Wald test

 ( 1)  [Inicio_Lactancia_Materna]0b.Nivel_Complejidad_Parto2#2.personal_atendio_parto2 = 0
 ( 2)  [Inicio_Lactancia_Materna]1.Nivel_Complejidad_Parto2#2.personal_atendio_parto2 = 0

       F(  2,  2969) =    0.00
            Prob > F =    0.9986
```

```
. svy, subpop (if incluidas==1 & nmiss==0): poisson Inicio_Lactancia_Mat i.Nivel_Complejidad_Parto2 i.Nivel_Complejidad_Parto2#personal_
> r  i.nivel_educativo_madre2 i.V190 i.Financiamiento_lugar_parto i.N_controles_prenatales4 i.orden_bebe i.V190 i.Tamaño_al_Nacer3, irr
(running poisson on estimation sample)

Survey: Poisson regression

Number of strata =   239                        Number of obs   =     21,038
Number of PSUs   = 3,209                        Population size = 9,707.2106
                                                Subpop. no. obs =      3,104
                                                Subpop. size    = 1,304.8767
                                                Design df       =      2,970
                                                F(22, 2949)     =       4.52
                                                Prob > F        =     0.0000

------------------------------------------------------------------------------------------------------------------
                                            |             Linearized
                   Inicio_Lactancia_Materna |        IRR   std. err.      t    P>|t|     [95% conf. interval]
--------------------------------------------+---------------------------------------------------------------------
                   Nivel_Complejidad_Parto2 |
                                   1º nivel |   1.095061   .0494725     2.01   0.045      1.00223    1.196491
                                            |
Nivel_Complejidad_Parto2#personal_atendio_parto2 |
                      2º y 3º nivel#Obstetra |   .9980285   .0373423    -0.05   0.958     .9274304    1.074001
                           1º nivel#Obstetra |   1.000225   .0408101     0.01   0.996     .9233228    1.083531
```

S2.2 Interaction test for the variable “place of residence”

```
.
. testparm i.Nivel_Complejidad_Parto2#i.V025

Adjusted Wald test

 ( 1)  [Inicio_Lactancia_Materna]1.Nivel_Complejidad_Parto2#1.V025 = 0

       F(  1,  2970) =    1.73
            Prob > F =    0.1884

.
end of do-file
```

```
. svy, subpop (if incluidas==1 & nmiss==0): poisson Inicio_Lactancia_Mat i.Nivel_Complejidad_Parto2 i.Nivel_Comple
> ivo_madre2 i.V190 i.Financiamiento_lugar_parto i.N_controles_prenatales4 i.orden_bebe i.V190 i.Tamaño_al_Nacer3,
(running poisson on estimation sample)

Survey: Poisson regression

Number of strata =   239                  Number of obs   =     21,038
Number of PSUs   = 3,209                  Population size = 9,707.2106
                                          Subpop. no. obs =      3,104
                                          Subpop. size    = 1,304.8767
                                          Design df       =      2,970
                                          F(21, 2950)     =       4.62
                                          Prob > F        =     0.0000
```

Inicio_Lactancia_Materna	IRR	Linearized std. err.	t	P>|t|	[95% conf.	interval]
Nivel_Complejidad_Parto2						
1º nivel	1.126467	.0421711	3.18	0.001	1.046742	1.212266
Nivel_Complejidad_Parto2#V025						
1º nivel#Rural	.9329395	.0492221	-1.32	0.188	.841251	1.034621

S2.3 Interaction test for the variable “healthcare facility financing”

```
. testparm i.Nivel_Complejidad_Parto2#i.Financiamiento_lugar_parto2

Adjusted Wald test

 ( 1)  [Inicio_Lactancia_Materna]1.Nivel_Complejidad_Parto2#1.Financiamiento_lugar_parto2 = 0

       F(  1,  2970) =    0.25
            Prob > F =    0.6175
```

```
. svy, subpop (if incluidas==1 & nmiss==0): poisson Inicio_Lactancia_Mat i.Nivel_Complejidad_Parto2 i.Nivel_Complejidad_Part
> l_nacer  i.nivel_educativo_madre2 i.V190 i.Financiamiento_lugar_parto i.N_controles_prenatales4 i.orden_bebe i.V190 i.Tama
(running poisson on estimation sample)

Survey: Poisson regression

Number of strata =   239                  Number of obs   =     21,038
Number of PSUs   = 3,209                  Population size = 9,707.2106
                                          Subpop. no. obs =      3,104
                                          Subpop. size    = 1,304.8767
                                          Design df       =      2,970
                                          F(21, 2950)     =       4.64
                                          Prob > F        =     0.0000
```

Inicio_Lactancia_Materna	IRR	Linearized std. err.	t	P>|t|	[95% conf.	interval]
Nivel_Complejidad_Parto2						
1º nivel	1.094038	.030812	3.19	0.001	1.03526	1.156152
Nivel_Complejidad_Parto2#Financiamiento_lugar_parto2						
1º nivel#ESSALUD	1.113738	.2401846	0.50	0.617	.7296949	1.699906
